# Towards a research renaissance: Empowering early career researchers through mentored AI use

**DOI:** 10.1371/journal.pbio.3003917

**Published:** 2026-08-11

**Authors:** Douglas R. Call, Mary Sanchez-Lanier, Ananth Kalyanaraman, Sascha H. Duttke

**Affiliations:** 1 Paul G Allen School for Global Health, Washington State University, Pullman, Washington, United States of America; 2 School of Molecular Biosciences, College of Veterinary Medicine, Washington State University, Pullman, Washington, United States of America; 3 School of Electrical Engineering and Computer Science, Washington State University, Pullman, Washington, United States of America

## Abstract

Generative AI is transforming the traditional apprenticeship-style approach to training researchers by lowering technical barriers. This Perspective argues that, when paired with careful mentoring, AI can help early career researchers engage more quickly in scientific inquiry.

## Teaser

Generative artificial intelligence (AI) is transforming the traditional apprenticeship-style approach to training researchers by lowering technical barriers. When paired with careful mentoring, AI can help early-career researchers engage more quickly in scientific inquiry.

Generative artificial intelligence (AI) might be the most consequential tool to enter the research laboratory in decades, and it is here to stay. Training researchers in its effective and ethical use is therefore no longer optional—it is critical for advancing science and workforce development. The risks associated with AI have been widely discussed, but AI use is also bringing immense opportunities, including earlier and more meaningful contributions to research questions by students and early career researchers (ECRs).

The transition of ECRs into independent researchers has historically been a rewarding but often lengthy process. Foundational knowledge and technical fluency are essential prerequisites for meaningful research engagement and, in specialized fields such as genomics or computational biology, researchers often spent months or years mastering programming and analytical methods before engaging in scientific discovery. As biological research becomes increasingly data-intensive, this training lag produces a growing entry burden, slower time-to-contribution, and notable dropout rates, negatively impacting the size and diversity of the scientific workforce [1–3]. This burden also hinders the pursuit of ‘out-of-the-box’ ideas by ECRs, prior to their being shaped by disciplinary convention.

**Fig 1:**
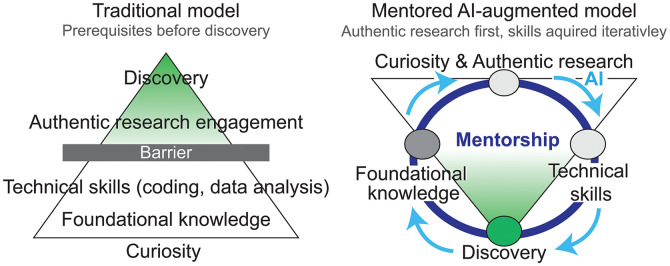
Generative AI inverts the traditional research training paradigm. In the traditional model, researchers must acquire foundational knowledge and technical skills such as programming before engaging in substantive research questions, resulting in delayed independence and high barriers to entry. In the AI-augmented model, researchers can engage in research at an early stage and acquire skills iteratively through a ‘learning-by-doing’ approach centered on curiosity and scientific inquiry. This shift accelerates training timelines, increases accessibility, and reframes skill acquisition from prerequisite to accompanying. Concurrently, the role of mentors evolves toward fostering critical thinking, experimental design, AI literacy, and rigorous validation. While this transformation introduces challenges related to over-reliance on AI and limited depth of understanding, it empowers early career researchers to engage in research questions, become productive, and learn faster.

AI tools are now positioned to reshape this paradigm (Fig 1). While foundational knowledge remains non-negotiable, AI has transformed how technical skills, particularly coding and data analysis, are acquired, acting as a bridge to earlier and more meaningful participation in research. Beyond gaining technical skills, AI can help a researcher understand a biological process, work through a statistical concept, brainstorm experimental designs, or orient themselves in an unfamiliar field; tasks that previously required a mentor. This fundamentally inverts the traditional training model: instead of front-loading technical prerequisites, ECRs can now acquire skills iteratively through learning-by-doing. Early evidence suggests this approach increases programming self-efficacy, motivation, productivity, and confidence [4,5], and empowers researchers to stretch beyond their traditional roles. AI can thus flatten the learning curve for technical prerequisites, placing problem-driven inquiry at the center of the research process. It also aligns the acquisition of technical knowledge more closely with how people naturally learn—iteratively, contextually, and out of curiosity. Importantly, this transition is not merely about pedagogy; by lowering barriers to entry in data-driven disciplines it has the potential to democratize participation in high-level scientific tasks, effectively expanding the research capacity of the entire field.

As with every tool, the utility of generative AI is inextricably linked to the user’s expertise. Because AI substantially improves performance and accelerates research when used skillfully, yet plateaus or actively misleads when users lack sufficient expertise or supervision [6,7], we believe that the mentored laboratory provides an ideal sandbox to learn effective and responsible use of AI. For example, mastering the nuances of various programming languages or data outputs often consumes cognitive bandwidth that could be reserved for hypothesis generation and data synthesis. By offloading routine technical tasks such as debugging scripts, critiquing initial drafts, and formatting outputs to AI, mentors and students can free-up time to focus on thinking about research questions, thereby shifting their attention up the abstraction ladder. This shift would also create space for more rigorous training of ECRs in experimental design, AI use, validation strategies, and scientific reasoning, thereby fostering deeper critical thinking. The result would be a more intellectually engaged training environment that accelerates both student development and the pace of discovery.

While AI cannot replace domain expertise or turn novices into experts, it can empower ECRs to more rapidly expand their capabilities. Agentic AI pushes this frontier even further by both helping with onboarding of new ECRs through agentic workflows and readily integrating new tools or concepts they develop back into the lab’s shared workflow. A researcher with conceptual mastery in one domain can leverage AI to translate that understanding into less familiar technical contexts; for instance, using AI-augmented analyses of publicly available data to directly test biological hypotheses, or seamlessly converting code from one programming language into another. Much like a musician applying music theory across different instruments, this AI-assisted horizontal knowledge transfer enables ECRs to bypass the steepest parts of the technical learning curve. Furthermore, ECRs are less shaped by disciplinary convention and more likely to ask the naïve but generative questions that spark serendipitous discovery. Coupled with the abundance of publicly available and often under-analyzed datasets, this democratization of technical skill can empower them to pursue curiosity-driven, out-of-the-box ideas that might previously have been dismissed as too technically demanding.

Realizing this potential, however, requires that AI serve as a bridge for technical gaps rather than as a surrogate for foundational knowledge, and that intellectual diversity be actively preserved. Because large language models are trained with broad, heterogeneous data, their outputs represent a probabilistic consensus that often lacks the nuance to distinguish high-quality evidence from outdated or retracted findings. Uncritical adoption may therefore drive convergence in thinking, quietly narrowing the very idea space that defines a renaissance [8,9]. AI is also likely to implicitly bias researchers toward data or methods that are widely accepted or computationally tractable, while underweighting complex, non-digitized, or ethically nuanced aspects of scientific inquiry [9], and it has blind spots for individual papers that challenge a field or paradigm shifts that are not yet statistically represented in its training data [8,9]. To counter these risks, AI must remain a scaffold for engaging with the literature, not a substitute for it. Specialized tools for literature synthesis can assist with the heavy lifting, but the responsibility for critical evaluation remains with the researcher. Mentors must train ECRs to recognize when AI gravitates toward the statistically likely rather than the biologically important—a distinction that requires prioritizing scientific logic over algorithmic probability. Indeed, as AI-assisted synthesis quietly infiltrates the scientific literature, careful reading of primary sources and data will be more, not less, important.

A central role for mentorship in the AI era is therefore teaching AI literacy: an understanding of model limitations, biases, and appropriate use cases [10]. A major challenge here is the risk of the ‘illusion of understanding’. Current AI systems optimize for coherence rather than correctness, producing outputs that may appear plausible even when they are incorrect [11]. AI-generated outputs are also fluent and authoritative by design, causing less-experienced users to overestimate their own comprehension [8] and bypass critical evaluation, evidence assessment, and analytical synthesis, a phenomenon increasingly being referred to as metacognitive laziness [12,13]. We believe that the laboratory provides a structured environment where expert guidance can counter these tendencies. When researchers learn to treat AI prompts as hypotheses—iterative starting points to be tested, refined, and scrutinized—they develop habits of mind that serve both effective AI use and scientific progress. Understanding that AI models are probabilistic predictors and not factual oracles is essential for avoiding the epistemic risk of confounding plausibility with proof [9]. Otherwise, using AI can quickly erode the depth of reasoning and compromise integrity and productivity. In the current AI era, verification (and not generation), becomes a core skill. This raises an apparent paradox: verifying AI output requires the very expertise ECRs are still developing [8]. We believe that mentorship can resolve this tension and foster the foundational knowledge from which independent verification can emerge.

Scientific progress has always traded procedural fluency for expanded capability. The calculator, for example, diminished reliance on manual computation but ultimately expanded what could be discovered. AI represents a similar inflection point, only at a far greater scale. Unlike a calculator, however, AI demands active verification rather than passive trust, and that distinction is precisely what makes the mentored environment so indispensable. Although integrating AI may initially seem daunting, its effective use relies on principles already deeply familiar to scientists: skepticism, validation, and iterative refinement. AI does not diminish scientific training, nor will it replace the scientist, but it can be used to recalibrate science toward what matters most—inquisitive reasoning, rigorous validation, nurturing the next generation of scientists, and translating curiosity into discovery.

## Abbreviations

AI: artificial intelligence
ECRs: early career researchers
